# A single dose of epidermicin NI01 is sufficient to eradicate MRSA from the nares of cotton rats

**DOI:** 10.1093/jac/dkw457

**Published:** 2016-12-13

**Authors:** Samantha Halliwell, Peter Warn, Abdul Sattar, Jeremy P. Derrick, Mathew Upton

**Affiliations:** 1 Faculty of Biology, Medicine and Health, University of Manchester, Manchester, UK; 2 Evotec (UK) Ltd, Manchester Science Park, Manchester, UK; 3 School of Biomedical & Healthcare Sciences, Plymouth University Peninsula Schools of Medicine and Dentistry, Plymouth, UK

## Abstract

**Objectives:** To investigate the efficacy of a potent novel antimicrobial protein of mass 6 kDa, epidermicin NI01, for eradicating the nasal burden of MRSA in a cotton rat (*Sigmodon hispidus*) model.

**Methods:** MRSA strain ATCC 43300 was used to establish a robust colonization of cotton rat nares. This model was used to evaluate the efficacy of topical 0.04% and 0.2% epidermicin NI01, administered twice daily for 3 days consecutively, and topical 0.8% epidermicin NI01 administered once, for reducing nasal MRSA burden. Control groups remained untreated or were administered vehicle only (0.5% hydroxypropylmethylcellulose) or 2% mupirocin twice daily for 3 days. The experiment was terminated at day 5 and MRSA quantitative counts were determined. Tissues recovered from animals treated with 0.2% epidermicin twice daily for 3 days were examined for histological changes.

**Results:** Mupirocin treatment resulted in a reduction in burden of log_10_ (log R) of 2.59 cfu/nares compared with vehicle (*P* < 0.0001). Epidermicin NI01 administered once at 0.8% showed excellent efficacy, resulting in a log R of 2.10 cfu/nares (*P* = 0.0004), which was equivalent to mupirocin. Epidermicin NI01 administered at 0.2% or 0.04% twice daily for 3 days did not have a significant impact on the tissue burden recovered from the nares. Mild to marked histological abnormalities were noted, but these were determined to be reversible.

**Conclusion:** A single dose of topical epidermicin NI01 was as effective as mupirocin administered twice daily for 3 days in eradication of MRSA from the nares of cotton rats. This justifies further development of epidermicin for this indication.

## Introduction

Nasal colonization by *Staphylococcus aureus* is a major risk factor for infection following surgery and other treatments such as renal dialysis.^1–4^ Accordingly, decolonization of *S. aureus* carriage reduces the risk of surgical site infections^1^^,^^5^ and can prevent MRSA infection in ICU patients.^4^

Currently, the most established decolonization programme involves treatment with mupirocin nasal ointment two or three times per day, for up to 10 days,^4^^,^^6^^,^^7^ but high-level mupirocin resistance,^8–11^ encoded by the *mupA* or *mupB* genes,^10^^,^^12^ is compromising this strategy. Mupirocin resistance is associated with failure to eradicate MRSA from the nares.^9^^,^^13^^,^^14^ Consequently, there is a pressing need to develop alternatives to mupirocin, and novel agents that allow reduced treatment regimens will be preferable and may lead to increased levels of compliance.

Bacteriocins are ribosomally synthesized antimicrobial peptides that have excellent potential for use in prevention or therapy of infections caused by a wide range of (multiresistant) bacteria.^15^^,^^16^ Epidermicin NI01, a novel class II bacteriocin, exhibits potent activity against a wide range of Gram-positive pathogens including MRSA,^17^ and protects *Galleria mellonella* larvae from MRSA infection.^18^ Having low toxicity, epidermicin has good potential for topical use. The cotton rat, with nares that structurally resemble those of humans, is a robust model for *S. aureus* nasal colonization, particularly when clearance over a longer term (up to 5 days) is required.^19^

The aim of the present study was to evaluate the effectiveness of single-dose and multi-dose epidermicin NI01 as an alternative agent for the nasal decolonization of MRSA in a cotton rat model.

## Materials and methods

### Ethics

All animal experiments were performed by Evotec (UK) Ltd, under UK Home Office License (PPL 40/3644) and with Ethics Committee approval, by technicians who had completed parts 1, 2 and 3 of the Home Office Personal License course.

### Bacterial strain

MRSA strain ATCC 43300 was used for all experiments. Frozen stocks were generated from log-phase cultures grown in nutrient broth containing 7.5% (w/v) NaCl (37°C, 300 rpm).

### Cotton rat husbandry

Female cotton rats (specific pathogen free) were supplied by S-A Ace, Inc. (Boyertown, PA, USA) and acclimatized for at least 14 days (91–125 g at the start of the infection model). Animals were housed in sterile, individually ventilated cages supplied with HEPA-filtered air with free access to food and sterile water and their sterile aspen chip bedding was changed as required. Room temperature was maintained at 22 ± 1°C with relative humidity at 50%–60%, a maximum background noise of 56 dB and a 12 h light/dark cycle with dawn/dusk phases.

### Infection of cotton rat nares with MRSA

MRSA cells (3.3 × 10^10^ cfu/mL in PBS) were passed 10 times through a 21G needle followed by similar treatment with a 23G needle to promote single cell suspension. Animals were briefly anaesthetized using inhaled anaesthesia (2.5% isoflurane/97.5% oxygen), held upright and 30 μL of inoculum was administered to the nares (15 μL per nostril). Animals were then held upright until fully conscious and were left for 24 h allowing bacteria to colonize the nares. Pilot studies confirmed that a high nasal burden of MRSA ATCC 43300 was successfully established using this protocol (data not shown).

### Administration of antimicrobial treatments to cotton rat nares

Epidermicin NI01 (>95% purity; Almac Ltd, Craigavon, UK) was suspended in 0.5% hydroxypropylmethylcellulose (HPMC) to generate 0.8%, 0.2% and 0.04% (w/v) stock solutions. Mupirocin (Sigma–Aldrich) was dissolved in 100% PEG400 at 2% (w/v). All solutions were stored at 4°C. At 19 h post-infection, treatments (15 μL per nostril, 30 μL per animal; Table 1) were administered intranasally to groups of up to seven animals under brief inhaled anaesthesia. Animals received: no treatment, a single treatment (0.8% epidermicin NI01) or multiple doses (0.2% epidermicin NI01, 0.04% epidermicin NI01 or 2% mupirocin) administered twice daily for 3 days consecutively (six doses). Vehicle control animals received 0.5% HPMC only, twice daily for 3 days. Animals were euthanized ∼36 h after the last treatment by pentobarbitone overdose and the nose area cleaned with 70% ethanol to eliminate external contamination by *S. aureus.* The nasal region was surgically excised, cut into small sections and homogenized in 3 mL of cold PBS using a Precellys bead beater. Serial dilutions were cultured onto Oxacillin Resistance Screening Agar Base (ORSAB) agar (Thermo Fisher, Basingstoke, UK) at 37°C for up to 72 h and cfu/nares determined. Changes in nasal MRSA burden were represented as log_10_ reduction (log R), compared with vehicle-only controls.

**Table 1:** Details of treatment schedules and nasal MRSA burdens in a cotton rat model used to examine the efficacy of epidermicin NI01 in nasal decolonization

Treatment	Regimen	No. of doses	Animals completing protocol	Mean cfu/nares	SD	Mean cfu/nares (log_10_)	Log R from vehicle control	Nostrils sterilized^a^
Untreated	—	—	4	6098	65415	3.79	–0.90	0
Vehicle (0.5% HPMC)	twice daily	6	7	766	6141	2.88	0.00	0
NI01 0.8%	once	1	5	6	177	0.78	2.10	3
NI01 0.2%	twice daily	6	6	699	5428	2.83	0.06	0
NI01 0.04%	twice daily	6	7	620	1628	2.79	0.09	0
Mupirocin 2%	twice daily	6	6	2	24	2.59	2.59	5

One out of five animals treated with 0.8% NI01 and two out of six animals treated with 0.2% NI01 had 1–4 colonies recovered on plating out 0.1 mL of undiluted homogenate and the burdens are likely not significant.

a No colonies recovered when plating 0.1 mL of undiluted homogenate.

### Statistical analysis

Quantitative tissue burden data were analysed using the Kruskal–Wallis test (corrected for multiple comparisons) and compared with the vehicle-only control using StatsDirect version 2.7.8. *P* < 0.05 was taken to indicate a significant difference between groups.

### Histology study of cotton rat nasal cavities

Groups of three naive cotton rats were treated intranasally twice daily for three consecutive days with 0.5% HPMC (vehicle) or 0.2% epidermicin NI01 in 0.5% HPMC (15 μL per nostril, 30 μL per animal). Animals were euthanized ∼12 h after the final treatment. The nose area of each animal was cleaned with 70% ethanol before heads were degloved and fixed in 10% formal saline prior to decalcification, wax embedding, sectioning and staining with haematoxylin and eosin, for histological examination (Propath UK Ltd, Hereford UK). Histological severity grades at three different levels of the nasal cavity were represented as: minimal, mild, moderate, marked and severe.

## Results

### Reduction of nasal burden following treatment

A robust infection model was successfully established with high burdens of MRSA in the untreated and vehicle-treated control group (log_10_ 3.79 and 2.88 cfu/nares, respectively). Some reduction in burden was observed following treatment with vehicle only, most likely due to organisms being flushed from the nostrils. Consequently, the effect of all active treatments has been compared with animals treated with vehicle only.

**Figure 1.**
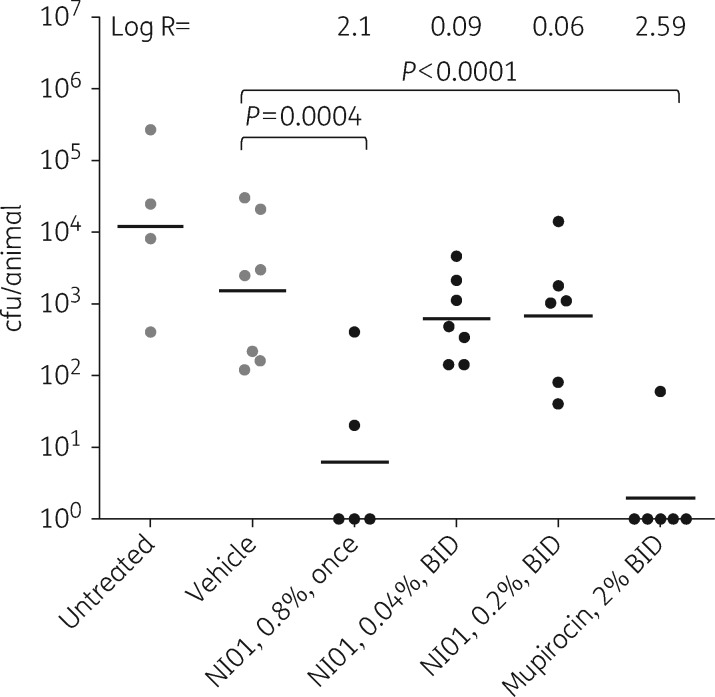
Scattergram of nasal burdens of MRSA in cotton rats following treatment with various concentrations of epidermicin NI01, vehicle only (0.5% HPMC) or mupirocin (2%). Limit of detection = 34 cfu/animal. BID, twice daily.

The effect of 0.8%, 0.2% and 0.04% epidermicin NI01 on cotton rat nasal MRSA burden was investigated in comparison with 2% mupirocin and animals treated with vehicle only (Table 1 and Figure 1). As anticipated, 2% mupirocin administered twice daily significantly (*P* < 0.0001) reduced the MRSA nasal burden by a log R of 2.59, the pathogen being completely eradicated in all but one case.

Interestingly, epidermicin NI01 administered only once at 0.8% demonstrated excellent efficacy. Treatment resulted in a significant log R of 2.10 (*P* = 0.0004), with eradication seen in 3/5 animals tested. The reduction in MRSA nasal burden was found to be statistically equivalent to that achieved following treatment with mupirocin. In contrast, epidermicin NI01 administered at 0.04% or 0.2% twice daily for 3 days did not reduce nasal MRSA burden (*P* ≥ 0.05).

### Tolerability of cotton rat nares to treatment with epidermicin NI01

Histological examination of treated animals, along with vehicle-treated animals, was performed to specifically identify any pathological changes in the nasal tissue. Mupirocin was not tested as this agent has been used previously in the cotton rat model without adverse effects.^20^ No abnormalities or inflammation were observed in vehicle-treated control animals. Treatment with 0.2% epidermicin NI01 for 3 days twice daily was associated with mild to marked epithelial abnormality, with a decreasing gradient of severity from anterior to posterior regions, but there were no severe effects (e.g. cytotoxicity, necrosis or presence of blood) noted.

## Discussion

Given increased resistance to nasal decolonization agents, including mupirocin, and the limited success in long-term prevention of MRSA carriage,^8–11^ new agents are required for this clinical indication. In the present study, we have used a robust and stringent cotton rat model of infection with colonization levels of ∼4 log_10_ cfu/nares achieved when sampling the entire nasal cavity. We have demonstrated that a single dose of 0.8% epidermicin NI01 is as effective at reducing MRSA nasal burden as six doses of 2% mupirocin. This finding is even more remarkable considering that MRSA burdens were measured 96 h after the dose was applied. Although we did not test a single dose of mupirocin, in a similar study Desbios *et al*.^7^ reported that such a treatment did not lead to a significant reduction in MRSA burden. It has previously been reported that a single dose of lysostaphin (0.5%) eradicated MRSA and MSSA carriage in cotton rats, with nasal loads determined 24 h after treatment.^20^ The histological effects of lysostaphin were not studied by Kokai-Kun *et al*.^20^ It is interesting to note that, in the same study, multiple doses (at 0.5% and 5%) of nisin, a potent antistaphylococcal bacteriocin, did not eradicate MRSA carriage from cotton rats,^20^ indicating superior performance of epidermicin over the only other bacteriocin investigated to date.

When considering frequency and duration of dosing, the efficacy of epidermicin observed in the current study represents a marked improvement in performance over the current drug of choice, and may facilitate a paradigm change in the treatment regimen for MRSA decolonization. It is also possible that a lower-concentration single dose with a superior formulation (as an alternative to the 0.5% HPMC used as a thickening agent in the current study) may be as effective. Alternatively, a combination treatment with lysostaphin, which has been shown to synergize with antimicrobial peptides in this model,^7^ might enhance efficacy.

Epidermicin NI01 administered at 0.2% or 0.04% for 3 days twice daily did not significantly reduce the MRSA burden recovered from the nares. This is likely to have been related to flushing of the epidermicin from the nares by increased mucus secretions, which would reduce exposure of MRSA to the peptide. It is possible that similar flushing occurred following multi-dose treatment with mupirocin, but historically mupirocin has not been reported as a cause of inflammation of nasal mucosa. Twice-daily treatments with epidermicin at 0.2% were associated with mild to marked histological defects and acute inflammation. All findings were considered to be reversible. Squamous metaplasia is generally considered to be a reversible adaptive change in response to irritation of the respiratory (or olfactory) epithelium, while the olfactory epithelium regenerates following injury, if the basal cells are intact. It is possible that use of a single dose of 0.8% epidermicin NI01 would reduce any histological changes due to the shorter exposure duration, though this was not examined in the current study.

This study represents the first demonstration of effective *in vivo* nasal MRSA decolonization by a type II bacteriocin and justifies further development of epidermicin NI01 as an alternative to mupirocin for the nasal decolonization of MRSA.
